# Supplementary material for: Nonuniversal impact of cholesterol on membranes mobility, curvature sensing and elasticity
Source: Nat Commun. 2023 Dec 11;14:8038. doi: 10.1038/s41467-023-43892-x (PMC10713574; doi:10.1038/s41467-023-43892-x)
Supplement: Supplementary file 1 — Supplementary Information [file 41467_2023_43892_MOESM1_ESM.pdf]

Supplementary Information for

Non-Universal Impact of Cholesterol on Membranes: Mobility, Curvature Sensing, and Elasticity

Matthias Pöhnl[1], Marius F.W. Trollmann[1,2], and Rainer A. Böckmann[1,2]

[1]Computational Biology, Department of Biology,
Friedrich-Alexander-Universität Erlangen-Nürnberg, Erlangen, Germany
[2]Erlangen National High-Perfomance Computing Center (NHR@FAU),
Erlangen, Germany
*rainer.boeckmann@fau.de

November 8, 2023

The PDF file includes

- Supplementary Discussion,
- Supplementary Figures 1 – 11,
- Supplementary Tables 1 – 6, and
- Supplementary References

Supplementary Discussion

Bending modulus derived from experiment and simulation

Recent ssNMR and NSE experiments, and MD simulations reported a substantial *increase* in the DOPC bending modulus upon addition of cholesterol[1,2]. Analysis of ssNMR assumes a homogeneous membrane, the local relaxation rate R_{1Z} is coupled to the lipid motion within the bilayer, yielding the relation $R_{1Z} \propto |S_{CD}|^2 \cdot K^{-3/2}$. Here, K is the elastic force constant for splay, twist and bend deformations[2]. In our simulations, however, $|S_{CD}|$ is increased and κ_b decreased if cholesterol is added to DOPC. Regarding timescales, the ^{2}H-NMR timescale of 10^{-5} s is above the relaxation timescales of cholesterol molecules between the membrane leaflets (between 0.1 µs and 1 µs), i.e. signals due to cholesterol distribution asymmetries will be averaged. Also in neutron spin echo (NSE) experiments, temporal relaxation rates could be directly related to the bending elasticity[2,3]. Similar to ssNMR, the underlying model does, however, not include changes in membrane composition in response to local curvature. Also, the NSE timescales of at most 100 ns do not cover major cholesterol redistribution (e.g. for all-atom i:DOPC^h: mean cholesterol displacement $\approx$ 1.2 nm and $\approx$ 0.04 flips per cholesterol within 100 ns) that we have shown to be key for membrane deformations.

Interestingly, also previous simulation studies at both all-atom and coarse-grained resolution reported an increase in the DOPC bending modulus upon addition of cholesterol[1,4]. One study[4] compared bending moduli derived from buckling simulations with those derived from the area compressibility, K_A and the membrane thickness h using polymer brush theory[5]: $\kappa_b = \frac{K_A}{24}(h - h_0)^2$. The latter derivation assumes one-component monolayers built of polymers that are held together by hydrophobic interactions, not allowing for exchange between each other. We have shown here that the conditions are not satisfied for membranes containing cholesterol. As earlier pointed out by Deserno and colleagues regarding buckling simulations, a more refined theory would need to include a coupling of the composition field to the locally varying curvature[6]. This is exactly what is observed here: The membrane softening by addition of cholesterol is coupled to the (locally) asymmetric and dynamic cholesterol composition.

The second study uses the real space fluctuation (RSF) analysis method to deduce the bending modulus from all-atom MD simulations. RSF determines the bending modulus from the splay modulus of lipid pairs. It assumes that each component of mixed membranes has its own κ_b, and assigns weights to each component with proportion to the number of occurences[7]. It appears that this empirical derivation breaks down for membranes containing more complex compositions with mobile molecules such as cholesterol, and that the here applied established Fourier-space based methods and a direct curvature analysis in real space (LFM) are beneficial for the analysis of complex membranes.

Supplementary Figures

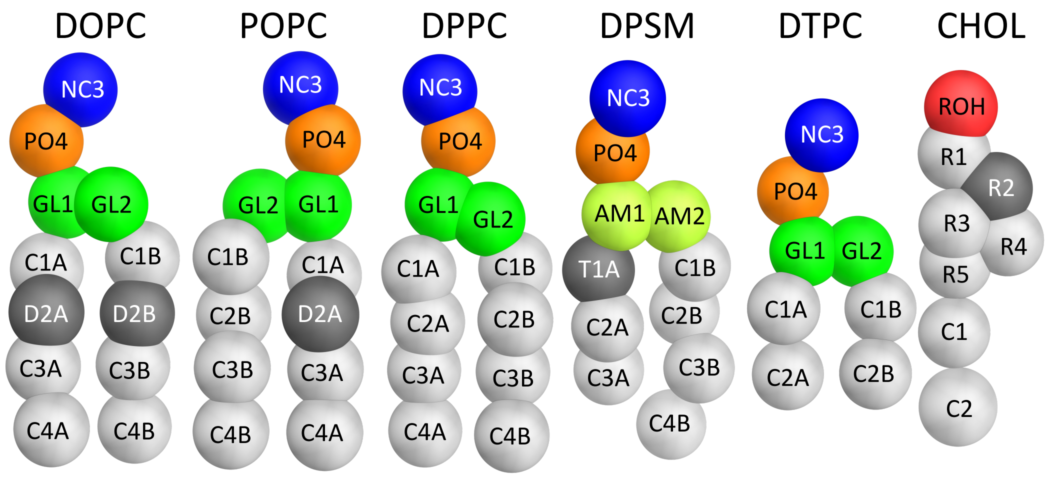

Figure 1: Lipids studied in bicelle and infinite systems in MARTINI coarse-grained representation, labeled with the respective bead names.

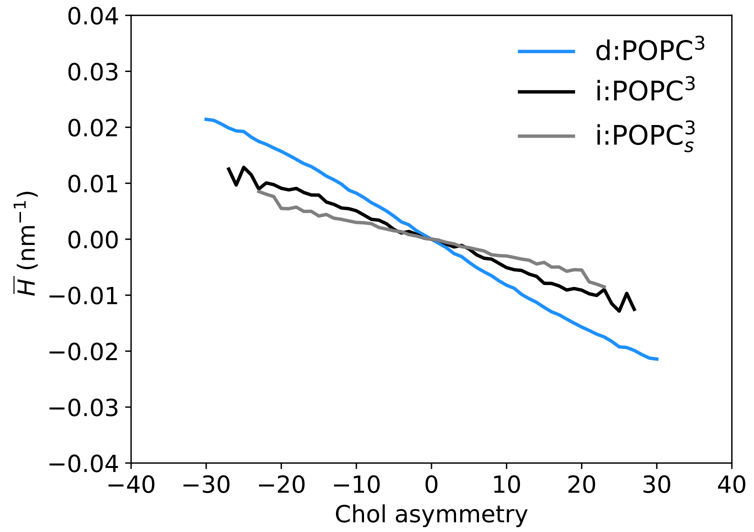

Figure 2: Coupling between membrane curvature and cholesterol distribution. Mean curvature values $\bar{H}$ are analyzed within circular domains of radius 7 nm and their time average is displayed as a function of cholesterol asymmetry for a bicelle system (*blue*), a small infinite bilayer (*gray*; boxlength ≈17 nm), and a large infinite bilayer (*black*; boxlength ≈28 nm) composed of POPC and ≈30 mol% cholesterol.

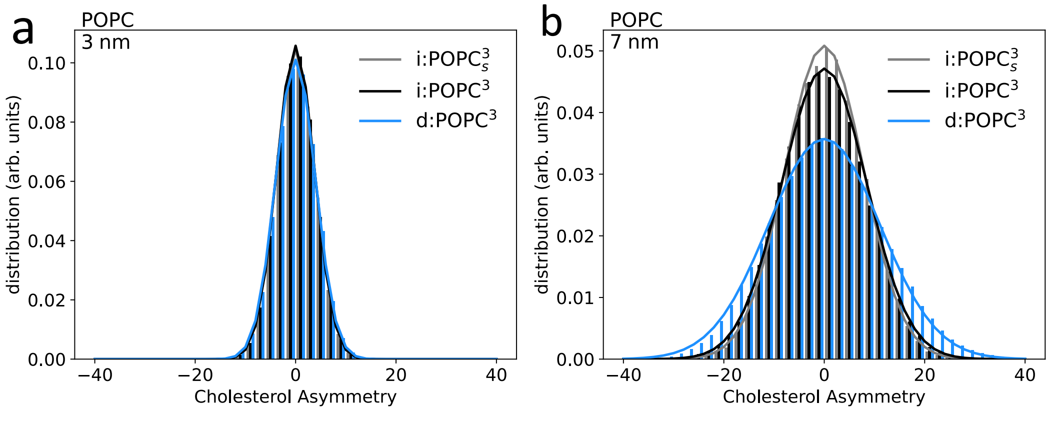

Figure 3: Cholesterol asymmetry for POPC systems Cholesterol asymmetries are analyzed for a bicelle system (*blue*), a small infinite bilayer (*gray*; boxlength ≈17 nm), and a large infinite bilayer (*black*; boxlength ≈28 nm) composed of POPC and ≈30 mol% cholesterol. Displayed are histograms of the cholesterol asymmetries within circular domains of **A** radius 3 nm and **B** radius 7 nm with a fit assuming a Gaussian distribution.

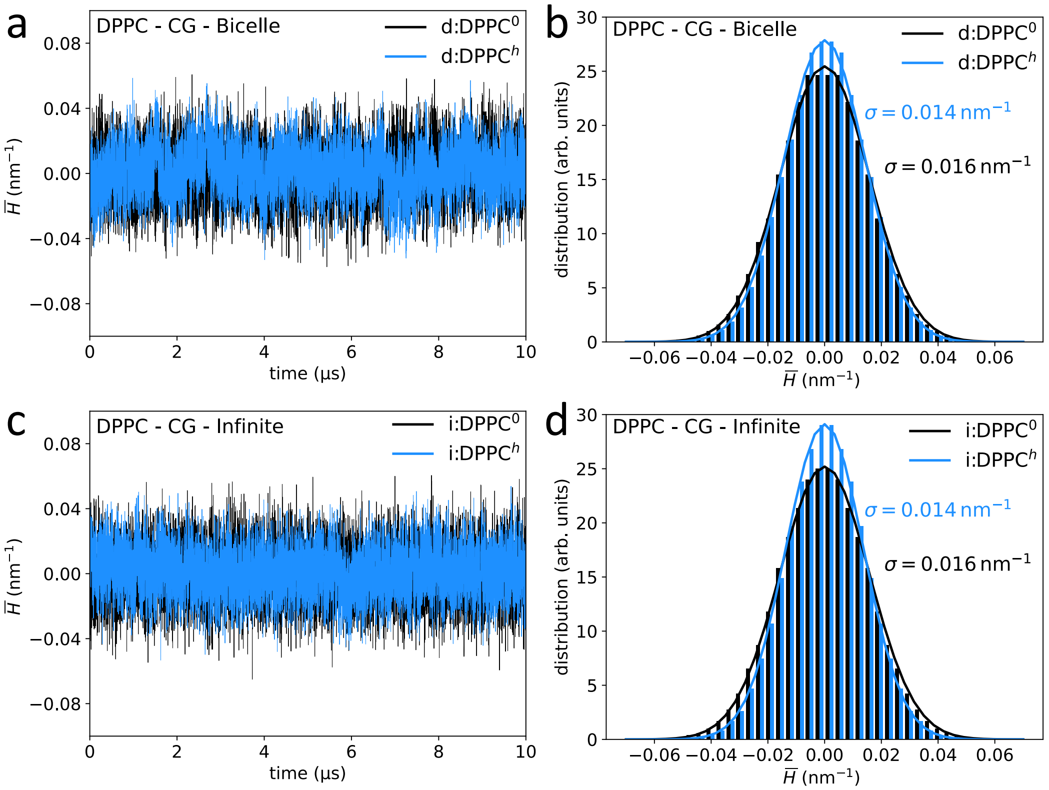

Figure 4: Membrane curvature for DPPC systems. Mean curvature values $\bar{H}$ within circular domains of radius 3 nm were analyzed for bicelle systems (**a,b**) and infinite systems (**c,d**) without (*black*) and with cholesterol (*blue*). Panels (**a,c**) show the time development and panels (**b,d**) histograms for the mean curvature with a fit assuming a Gaussian distribution.

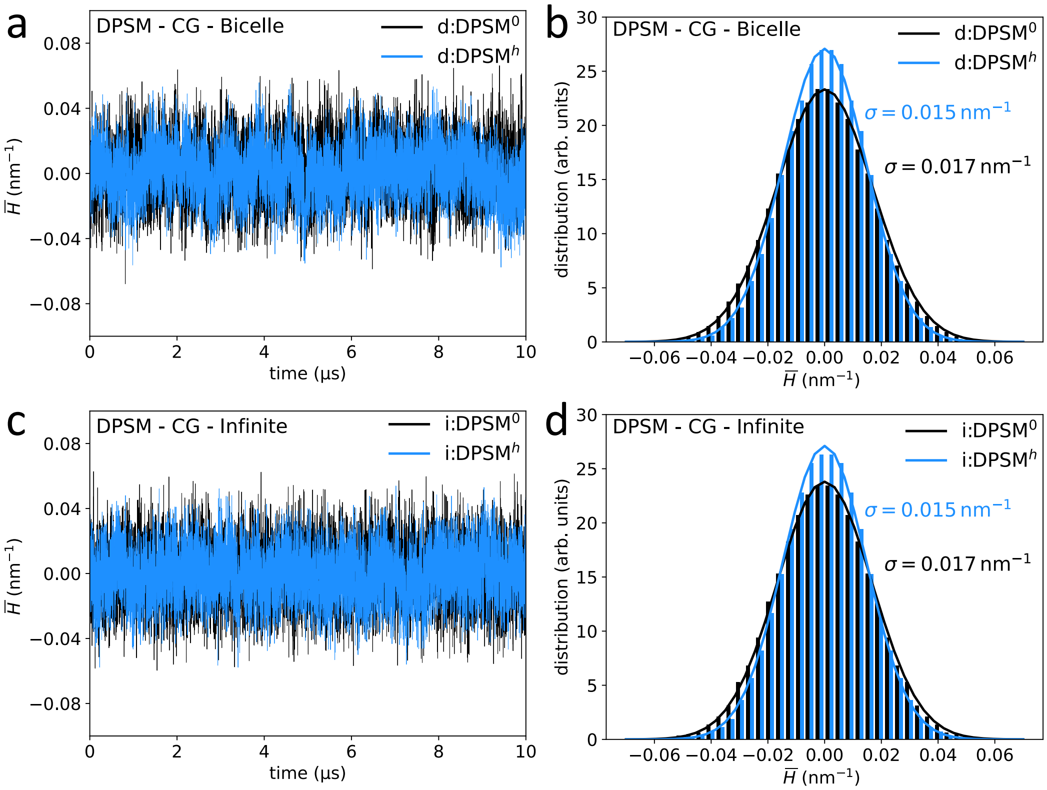

Figure 5: Membrane curvature for DPSM systems. Mean curvature values $\bar{H}$ within circular domains of radius 3 nm were analyzed for bicelle systems (**a,b**) and infinite systems (**c,d**) without (*black*) and with cholesterol (*blue*). Panels (**a,c**) show the time development and panels (**b,d**) histograms for the mean curvature with a fit assuming a Gaussian distribution.

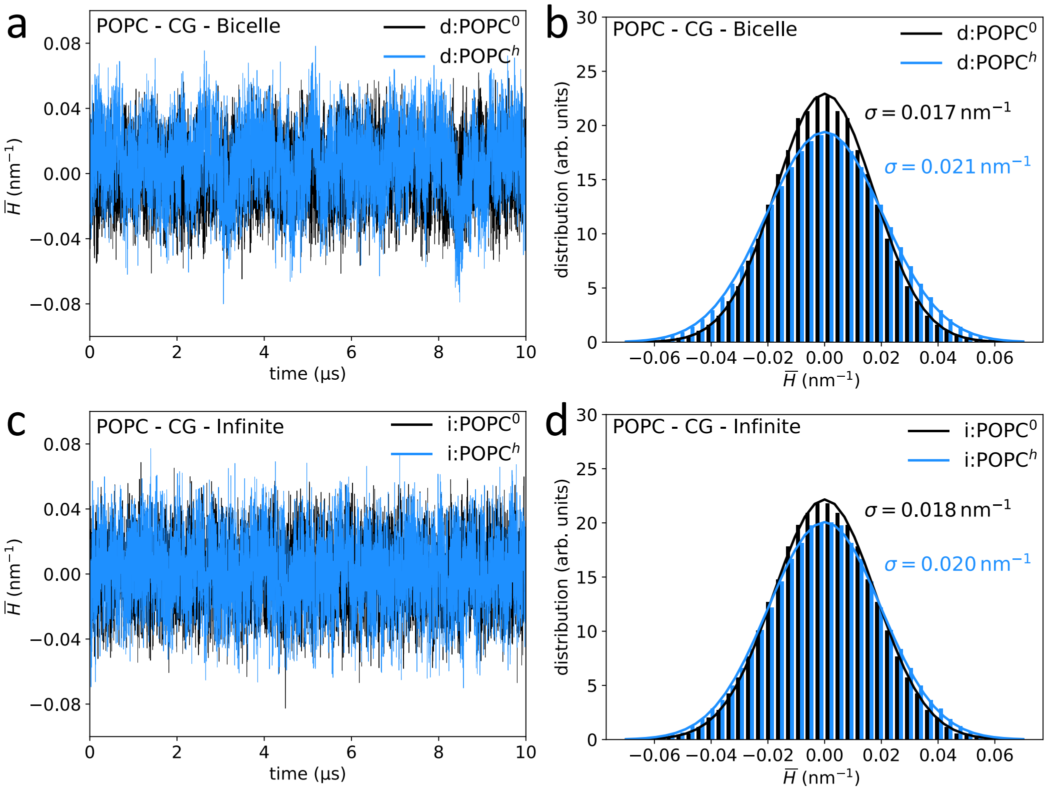

Figure 6: Membrane curvature for POPC systems. Mean curvature values $\bar{H}$ within circular domains of radius 3 nm were analyzed for bicelle systems (**a,b**) and infinite systems (**c,d**) without (*black*) and with cholesterol (*blue*). Panels (**a,c**) show the time development and panels (**b,d**) histograms for the mean curvature with a fit assuming a Gaussian distribution.

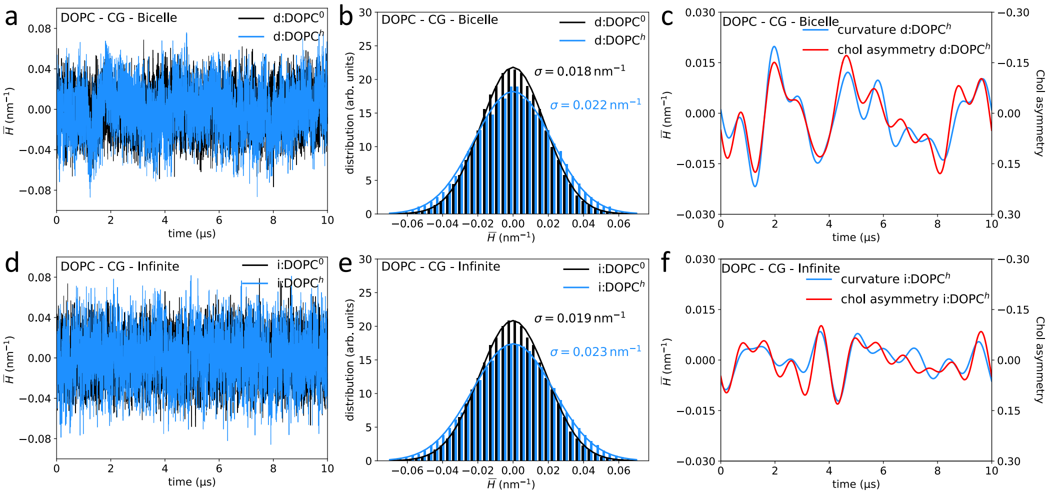

Figure 7: Membrane curvature for DOPC systems. Mean curvature values $\bar{H}$ within circular domains of radius 3 nm were analyzed for bicelle systems (**a,b,c**) and infinite systems (**d,e,f**) without (*black*) and with cholesterol (*blue*). Panels (**a,d**) show the time development and panels (**b,e**) histograms for the mean curvature with a fit assuming a Gaussian distribution. Panels (**c,f**) show the low-pass filtered mean curvature values (*blue*) together with the asymmetric distribution of cholesterol between the two leaflets within the analyzed circular domain (*red*; difference in the number of cholesterol molecules between the upper and lower leaflets, normalized to the average number within one leaflet).

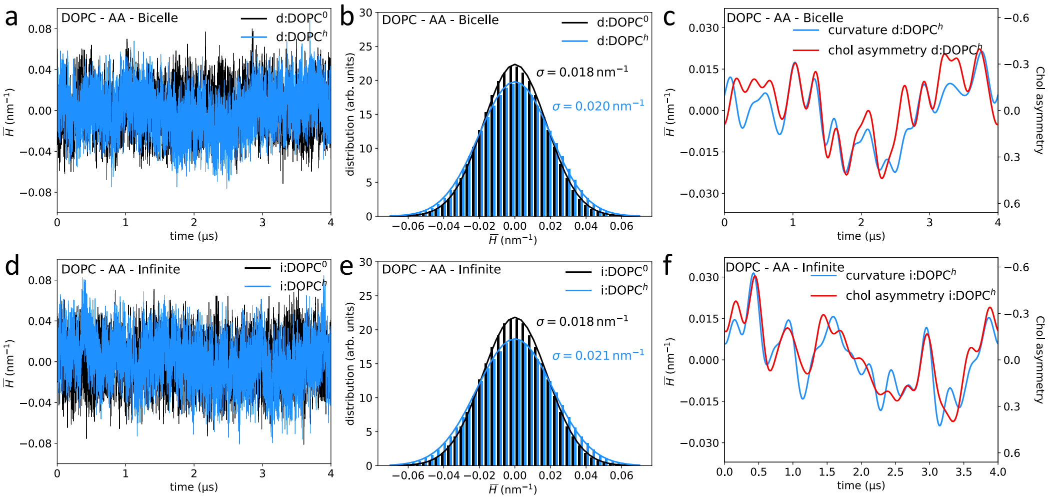

Figure 8: Membrane curvature for DOPC systems at all-atom resolution. Mean curvature values $\bar{H}$ within circular domains of radius 3 nm were analyzed for bicelle systems (**a,b,c**) and infinite systems (**d,e,f**) without (*black*) and with cholesterol (*blue*) at all-atom resolution. Panels (**a,d**) show the time development and panels (**b,e**) histograms for the mean curvature with a fit assuming a Gaussian distribution. Panels (**c,f**) show the low-pass filtered mean curvature values (*blue*) together with the asymmetric distribution of cholesterol between the two leaflets within the analyzed circular domain (*red*; difference in the number of cholesterol molecules between the upper and lower leaflets, normalized to the average number within one leaflet).

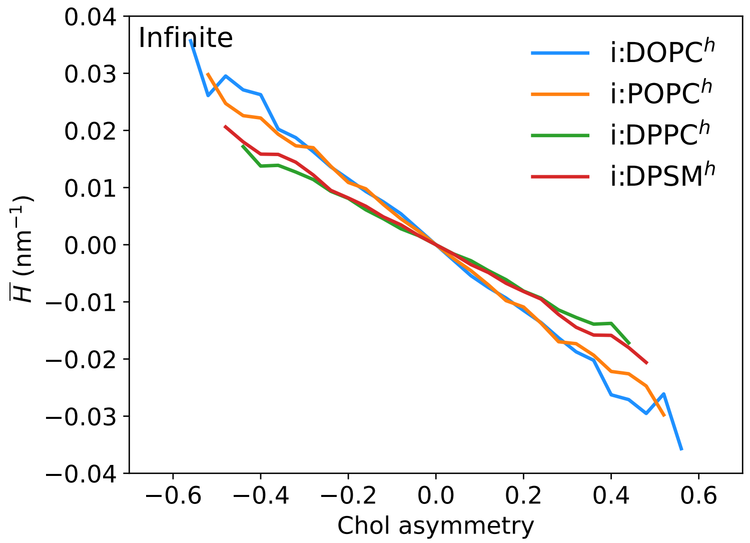

Figure 9: Coupling between membrane curvature and cholesterol distribution. Mean curvature values $\bar{H}$ are analyzed within circular domains of radius 3 nm and their time average is displayed as a function of cholesterol asymmetry for infinite bilayers at CG resolution.

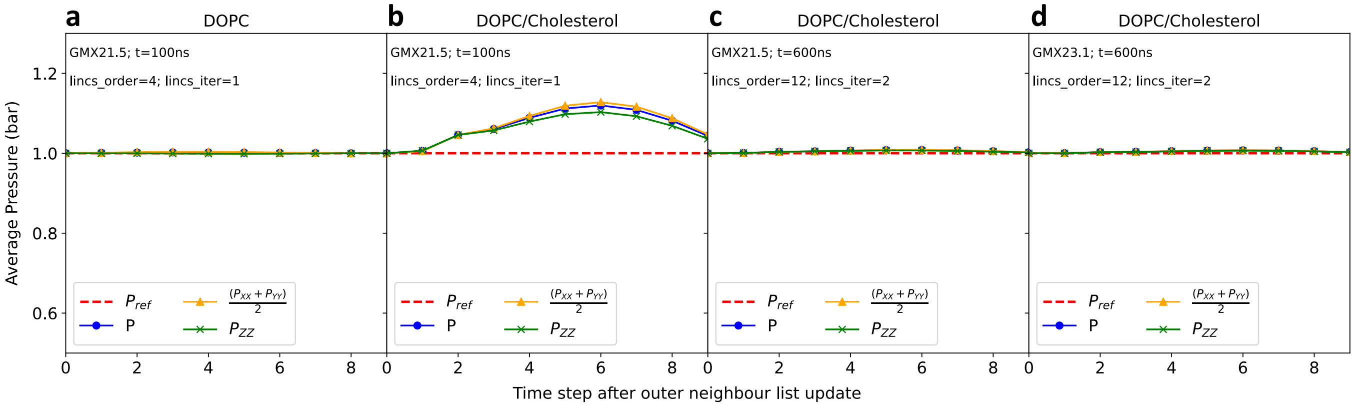

Figure 10: Pressure in membrane systems. Average pressure tensor components for each integration step between neighbour list updates for (a) DOPC and (b-d) DOPC/Cholesterol membranes, spanning a size of $110\,nm \times 110\,nm$. Shown are the curves for the reference pressure (*red dotted line*), the scalar pressure (*blue*), the lateral pressure (*orange*), and the normal pressure (*green*). Simulation parameters were selected inspired by the recommendations of Kim *et al.* (*8*), which include `nstlist=10`, `rlist=1.5nm`, `nsttcouple=10`, `nstpcouple=10`, and `verlet-buffer-tolerance=-1`. Notably, for the binary DOPC/cholesterol membrane, the LINCS parameters need to be adapted to minimize deviations from the reference pressure. The modified LINCS parameters were suggested earlier to avoid artificial temperature differences between cholesterol and phospholipids (*9*).

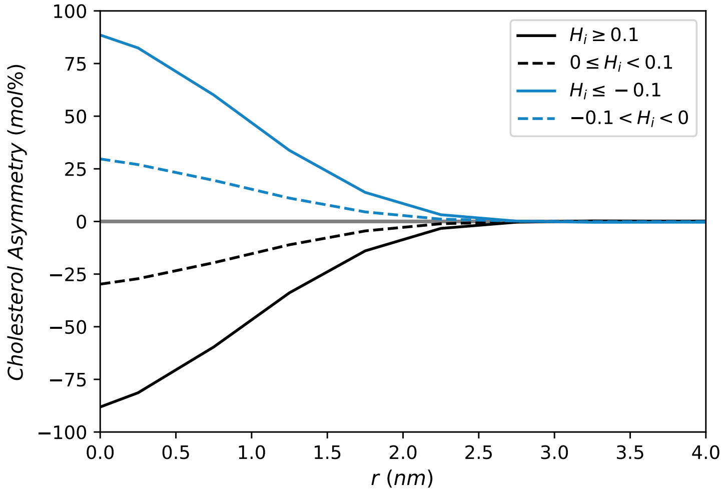

Figure 11: Coupling between local membrane curvature and cholesterol distribution. The graphs show the cholesterol asymmetry as a function of distance r from grid points of defined curvature, analyzed for an all-atom simulation of an infinite DOPC membrane (40 mol% cholesterol) at a temperature of 320 K (i:DOPC^h:AA). For each leaflet, (local) curvature and cholesterol molecules were mapped onto a grid with a constant grid spacing of approximately 0.3 nm. Cholesterol assignment to a leaflet was determined based on a cutoff distance of 1 nm, considering the distance between the oxygen atom of cholesterol and the phosphorus atom of DOPC. Asymmetry was determined by subtracting the number of cholesterol between opposing grid points in each monolayer and dividing it by the (globally) expected number of cholesterol per grid point. The different lines were obtained by summarizing grid points with specific values for the average local curvature H_i between both leaflets.

Supplementary Tables

System	Lipid bicelle disc or infinite bilayer (#(CHOL))/#(lipids)	Central bicelle domain: (#(CHOL))/#(lipids)	T in K	Sim.time in μs	Box in nm
d:DPPC	0-436 / 850-730	0-248 / 490-452	320	10	32
i:DPPC	0-1410 / 2450-2116	–	320	10	27
d:DPSM	0-488 / 860-732	0-249 / 499-456	320	10	32
i:DPSM	0-1410 / 2450-2116	–	320	10	27
d:POPC	0-444 / 798-668	0-208 / 459-414	320	10	32
i:POPC	0-1280 / 2312-1920	–	320	10	27
d:DOPC	0-422 / 766-632	0-200 / 441-376	320	10	32
i:DOPC	0-1216 / 2312-1824	–	320	10	27
i:DOPC: 55 nm	0-4866 / 9248-7300	–	320	10	55
i:DOPC:110 nm	0-19468 / 36992-29202	–	320	10/13	110
i:DPPC: 17 nm	578 / 868	–	320	6	18
i:DPPC:noflip	578 / 868	–	320	6	18
i:DPPC:restr.	578 / 868	–	320	6	18
i:DOPC: 17 nm	512 / 768	–	320	6	18
i:DOPC:noflip	512 / 768	–	320	6	18
i:DOPC:restr.	512 / 768	–	320	6	18
d:POPC^0:AA	798	466	320	4	29
d:POPC^2:AA	186 / 748	98 / 440	320	4	29
d:DOPC^0:AA	766	446	320	4	29
d:DOPC^h:AA	422 / 632	200 / 376	320	4	30
i:DPPC^0:AA	2450	–	330	4	27
i:DPPC^h:AA	1410 / 2116	–	330	4	27
i:DOPC^0:AA	1824	–	320	4	25
i:DOPC^h:AA	1216 / 1824	–	320	4	28
i:DOPC$_s^0$:AA	882	–	298/310	4	17
i:DOPC$_s^h$:AA	490 / 734	–	298/310	4	17

Table 1: Coarse-grained and all-atom (AA) simulation systems. Both lipid bicelles (d:) and infinite lipid bilayer systems (i:) were studied for varying cholesterol content (five different concentrations for most coarse-grained systems). Additionally, the (equilibrated) lipid composition within the central circular bicelle domain of radius 7 nm, the temperature, the simulation time, and the lateral box length are provided. The total bicelles including the bicelle disc and the rim domain have an overall radius of 13.5 nm[10].

System	T in K	d in nm	A_l in nm^2	P_2	D in 10^{-6}cm^2 s^{-1}
d:DPPC^0	320	3.99 ± 0.01	0.63 ± 0.01	0.61 ± 0.01	0.72 ± 0.01
d:DPPC^1	320	4.09 ± 0.01	$0.59 \pm 0.01/0.45 \pm 0.01$	0.65 ± 0.01	$0.59 \pm 0.01/0.71 \pm 0.02$
d:DPPC^2	320	4.19 ± 0.01	$0.55 \pm 0.01/0.44 \pm 0.01$	0.70 ± 0.01	$0.45 \pm 0.01/0.50 \pm 0.02$
d:DPPC^3	320	4.28 ± 0.01	$0.51 \pm 0.01/0.43 \pm 0.01$	0.75 ± 0.01	$0.29 \pm 0.01/0.31 \pm 0.01$
d:DPPC^h	320	4.32 ± 0.01	$0.48 \pm 0.01/0.41 \pm 0.01$	0.79 ± 0.01	$0.18 \pm 0.01/0.19 \pm 0.01$
d:POPC^0	320	3.78 ± 0.01	0.68 ± 0.01	0.53 ± 0.01	0.79 ± 0.01
d:POPC^0:AA	320	3.75 ± 0.01	0.67 ± 0.01	0.14 ± 0.01	0.16 ± 0.01
d:POPC^1	320	3.84 ± 0.01	$0.65 \pm 0.01/0.49 \pm 0.01$	0.55 ± 0.01	$0.68 \pm 0.01/0.87 \pm 0.02$
d:POPC^2	320	3.89 ± 0.01	$0.61 \pm 0.01/0.48 \pm 0.01$	0.57 ± 0.01	$0.56 \pm 0.01/0.71 \pm 0.02$
d:POPC^2:AA	320	3.98 ± 0.01	$0.61 \pm 0.01/0.47 \pm 0.01$	0.19 ± 0.01	$0.13 \pm 0.01/0.16 \pm 0.01$
d:POPC^3	320	3.97 ± 0.01	$0.56 \pm 0.01/0.47 \pm 0.01$	0.61 ± 0.01	$0.40 \pm 0.01/0.49 \pm 0.01$
d:POPC^h	320	4.00 ± 0.01	$0.54 \pm 0.01/0.46 \pm 0.01$	0.63 ± 0.01	$0.32 \pm 0.01/0.40 \pm 0.01$
d:DOPC^0	320	3.66 ± 0.01	0.71 ± 0.01	0.47 ± 0.01	0.86 ± 0.01
d:DOPC^0:AA	320	3.73 ± 0.01	0.70 ± 0.01	0.11 ± 0.01	0.17 ± 0.01
d:DOPC^1	320	3.70 ± 0.01	$0.68 \pm 0.01/0.51 \pm 0.01$	0.49 ± 0.01	$0.75 \pm 0.01/0.98 \pm 0.03$
d:DOPC^2	320	3.74 ± 0.01	$0.65 \pm 0.01/0.51 \pm 0.01$	0.50 ± 0.01	$0.63 \pm 0.01/0.83 \pm 0.02$
d:DOPC^3	320	3.79 ± 0.01	$0.61 \pm 0.01/0.50 \pm 0.01$	0.52 ± 0.01	$0.50 \pm 0.01/0.66 \pm 0.02$
d:DOPC^h	320	3.82 ± 0.01	$0.57 \pm 0.01/0.49 \pm 0.01$	0.54 ± 0.01	$0.38 \pm 0.01/0.49 \pm 0.01$
d:DOPC^h:AA	320	4.01 ± 0.01	$0.60 \pm 0.01/0.47 \pm 0.01$	0.15 ± 0.01	$0.12 \pm 0.01/0.13 \pm 0.01$
d:DPSM^0	320	3.67 ± 0.01	0.63 ± 0.01	0.65 ± 0.01	0.81 ± 0.01
d:DPSM^1	320	3.77 ± 0.01	$0.59 \pm 0.01/0.44 \pm 0.01$	0.68 ± 0.01	$0.65 \pm 0.01/0.77 \pm 0.02$
d:DPSM^2	320	3.88 ± 0.01	$0.55 \pm 0.01/0.44 \pm 0.01$	0.72 ± 0.01	$0.47 \pm 0.01/0.52 \pm 0.01$
d:DPSM^3	320	3.99 ± 0.01	$0.51 \pm 0.01/0.42 \pm 0.01$	0.76 ± 0.01	$0.30 \pm 0.01/0.31 \pm 0.01$
d:DPSM^h	320	4.05 ± 0.01	$0.48 \pm 0.01/0.41 \pm 0.01$	0.80 ± 0.01	$0.18 \pm 0.01/0.18 \pm 0.01$

Table 2: Temperature T, membrane thickness d, area per lipid A_l, averaged tail order parameter P_2 and diffusion coefficient D, averaged over all lipids within the bicelle central analysis domain (radius 7 nm). Values for the area per lipid A_l and the diffusion coefficient D are calculated separately for the studied phospholipids and cholesterol. Errors are given as standard errors of the mean employing block averaging (at least $N = 10$ independent blocks).

**System**	T in K	d in nm	A_l in nm^2	P_2	D in 10^{-6} cm^2 s^{-1}
i:DPPC^0	320	4.00 ± 0.01	0.63 ± 0.01	0.61 ± 0.01	0.85 ± 0.01
i:DPPC^0:AA	330	3.87 ± 0.01	0.62 ± 0.01	0.18 ± 0.01	0.23 ± 0.01
i:DPPC^1	320	4.10 ± 0.01	$0.59 \pm 0.01/0.45 \pm 0.01$	0.66 ± 0.01	$0.71 \pm 0.01/0.84 \pm 0.01$
i:DPPC^2	320	4.20 ± 0.01	$0.55 \pm 0.01/0.44 \pm 0.01$	0.71 ± 0.01	$0.54 \pm 0.01/0.61 \pm 0.01$
i:DPPC^3	320	4.29 ± 0.01	$0.50 \pm 0.01/0.42 \pm 0.01$	0.76 ± 0.01	$0.36 \pm 0.01/0.39 \pm 0.01$
i:DPPC^h	320	4.34 ± 0.01	$0.47 \pm 0.01/0.40 \pm 0.01$	0.81 ± 0.01	$0.20 \pm 0.01/0.22 \pm 0.01$
i:DPPC^h:AA	330	4.61 ± 0.01	$0.44 \pm 0.01/0.39 \pm 0.01$	0.34 ± 0.01	$0.08 \pm 0.01/0.09 \pm 0.01$
i:POPC^0	320	3.81 ± 0.01	0.67 ± 0.01	0.54 ± 0.01	0.93 ± 0.01
i:POPC^1	320	3.86 ± 0.01	$0.64 \pm 0.01/0.48 \pm 0.01$	0.56 ± 0.01	$0.79 \pm 0.01/0.99 \pm 0.01$
i:POPC^2	320	3.93 ± 0.01	$0.60 \pm 0.01/0.48 \pm 0.01$	0.59 ± 0.01	$0.63 \pm 0.01/0.78 \pm 0.01$
i:POPC^3	320	3.98 ± 0.01	$0.56 \pm 0.01/0.47 \pm 0.01$	0.61 ± 0.01	$0.50 \pm 0.01/0.61 \pm 0.01$
i:POPC^4	320	3.99 ± 0.01	$0.55 \pm 0.01/0.47 \pm 0.01$	0.62 ± 0.01	$0.48 \pm 0.01/0.58 \pm 0.01$
i:POPC^h	320	4.06 ± 0.01	$0.51 \pm 0.01/0.45 \pm 0.01$	0.65 ± 0.01	$0.34 \pm 0.01/0.40 \pm 0.01$
i:DOPC^0	320	3.69 ± 0.01	0.70 ± 0.01	0.48 ± 0.01	1.00 ± 0.01
i:DOPC^0:AA	298	3.82 ± 0.01	0.68 ± 0.01	0.12 ± 0.01	0.10 ± 0.01
i:DOPC^0:AA	310	3.80 ± 0.01	0.69 ± 0.01	0.12 ± 0.01	0.15 ± 0.01
i:DOPC^0:AA	320	3.77 ± 0.01	0.70 ± 0.01	0.11 ± 0.01	0.24 ± 0.01
i:DOPC^1	320	3.73 ± 0.01	$0.67 \pm 0.01/0.50 \pm 0.01$	0.50 ± 0.01	$0.86 \pm 0.01/1.12 \pm 0.01$
i:DOPC^2	320	3.78 ± 0.01	$0.63 \pm 0.01/0.50 \pm 0.01$	0.51 ± 0.01	$0.70 \pm 0.01/0.89 \pm 0.01$
i:DOPC^3	320	3.83 ± 0.01	$0.59 \pm 0.01/0.49 \pm 0.01$	0.54 ± 0.01	$0.53 \pm 0.01/0.67 \pm 0.01$
i:DOPC^h	320	3.88 ± 0.01	$0.54 \pm 0.01/0.47 \pm 0.01$	0.56 ± 0.01	$0.38 \pm 0.01/0.48 \pm 0.01$
i:DOPC^h:AA	298	4.40 ± 0.01	$0.51 \pm 0.01/0.41 \pm 0.01$	0.21 ± 0.01	$0.08 \pm 0.01/0.08 \pm 0.01$
i:DOPC^h:AA	310	4.34 ± 0.01	$0.52 \pm 0.01/0.42 \pm 0.01$	0.20 ± 0.01	$0.11 \pm 0.01/0.11 \pm 0.01$
i:DOPC^h:AA	320	4.02 ± 0.01	$0.59 \pm 0.01/0.46 \pm 0.01$	0.15 ± 0.01	$0.14 \pm 0.01/0.15 \pm 0.01$
i:DPSM^0	320	3.69 ± 0.01	0.62 ± 0.01	0.65 ± 0.01	0.93 ± 0.01
i:DPSM^1	320	3.78 ± 0.01	$0.58 \pm 0.01/0.44 \pm 0.01$	0.68 ± 0.01	$0.76 \pm 0.01/0.88 \pm 0.01$
i:DPSM^2	320	3.89 ± 0.01	$0.54 \pm 0.01/0.43 \pm 0.01$	0.73 ± 0.01	$0.56 \pm 0.01/0.62 \pm 0.01$
i:DPSM^3	320	4.00 ± 0.01	$0.50 \pm 0.01/0.42 \pm 0.01$	0.77 ± 0.01	$0.36 \pm 0.01/0.38 \pm 0.01$
i:DPSM^h	320	4.07 ± 0.01	$0.46 \pm 0.01/0.40 \pm 0.01$	0.81 ± 0.01	$0.21 \pm 0.01/0.21 \pm 0.01$

Table 3: Temperature T, membrane thickness d, area per lipid A_l, averaged tail order parameter P_2 and diffusion coefficient D, averaged over all lipids within the infinite lipid bilayer systems. Values for the area per lipid A_l and the diffusion coefficient D are calculated separately for the studied phospholipids and cholesterol. Errors are given as standard errors of the mean employing block averaging (at least $N = 10$ independent blocks).

System	T in K	κ_b^{u} in $k_{\mathrm{B}}T$	$\kappa_\theta^{\mathrm{u}}$ in $k_{\mathrm{B}}Tnm^{-2}$	q_c in nm^{-1}	κ_b^{o} in $k_{\mathrm{B}}T$	κ_b^{R} in $k_{\mathrm{B}}T$
i:DPPC^0	320	30.70 [30.36; 31.04]	31.31 [30.72; 31.92]	5.30 [5.05; 5.59]	27.46 [27.21; 27.71]	24.08 ± 0.03
i:DPPC^0:AA	330	28.24 [26.96; 29.64]	11.88 [11.35; 12.47]	$-^*$	29.90 [29.69; 30.11]	29.98 ± 0.02
i:DPPC^1	320	28.67 [28.36; 28.99]	34.47 [33.72; 35.24]	4.72 [4.53; 4.94]	25.70 [25.44; 25.96]	33.33 ± 0.22
i:DPPC^2	320	29.06 [28.68; 29.45]	37.51 [36.59; 38.47]	4.46 [4.29; 4.66]	26.15 [25.77; 26.53]	44.60 ± 0.15
i:DPPC^3	320	34.35 [33.91; 34.80]	38.96 [38.12; 39.81]	4.28 [4.15; 4.43]	31.48 [30.99; 31.98]	62.08 ± 0.24
i:DPPC^h	320	43.71 [43.18; 44.25]	38.75 [38.07; 39.44]	4.31 [4.18; 4.45]	41.90 [41.41; 42.40]	82.15 ± 0.26
i:DPPC^0:AA	330	106.3 [101.1; 112.1]	19.04 [18.87; 19.21]	$-^*$	111.2 [109.3; 113.2]	156.7 ± 0.31
i:POPC^0	320	22.05 [21.82; 22.27]	34.32 [33.47; 35.21]	4.31 [4.16; 4.47]	20.05 [19.88; 20.23]	17.62 ± 0.02
i:POPC^1	320	19.34 [19.13; 19.56]	38.13 [36.93; 39.40]	3.95 [3.83; 4.08]	17.59 [17.36; 17.82]	21.51 ± 0.04
i:POPC^2	320	17.96 [17.76; 18.16]	41.56 [40.08; 43.14]	3.78 [3.67; 3.90]	16.08 [15.87; 16.29]	24.28 ± 0.04
i:POPC^3	320	17.47 [17.27; 17.67]	44.60 [42.87; 46.45]	3.64 [3.54; 3.76]	15.76 [15.55; 15.98]	27.80 ± 0.04
i:POPC^4	320	17.38 [17.13; 17.65]	44.87 [42.47; 47.51]	3.62 [3.49; 3.78]	15.65 [15.38; 15.94]	28.98 ± 0.05
i:POPC^h	320	16.87 [16.67; 17.07]	54.60 [51.81; 57.63]	3.32 [3.24; 3.41]	15.01 [14.75; 15.28]	36.80 ± 0.06
i:DOPC^0	320	18.82 [18.59; 19.05]	35.26 [33.79; 36.83]	4.19 [3.95; 4.47]	17.16 [17.01; 17.31]	15.16 ± 0.02
i:DOPC^0:AA	298	24.14 [23.12; 25.25]	21.65 [20.64; 22.72]	8.75 [6.60; 19.2]	23.81 [23.42; 24.21]	17.92 ± 0.01
i:DOPC^0:AA	310	23.07 [21.86; 24.42]	22.03 [20.38; 23.95]	7.35 [5.40; 22.1]	22.22 [21.85; 22.59]	17.17 ± 0.01
i:DOPC^0:AA	320	21.65 [21.10; 22.24]	22.29 [21.29; 23.37]	8.38 [6.55; 14.2]	21.04 [20.09; 21.22]	16.48 ± 0.02
i:DOPC^1	320	16.03 [15.89; 16.17]	41.63 [40.12; 43.23]	3.70 [3.59; 3.81]	14.76 [14.61; 14.91]	17.70 ± 0.02
i:DOPC^2	320	14.38 [14.24; 14.52]	46.46 [44.39; 48.69]	3.55 [3.45; 3.65]	13.29 [13.13; 13.45]	18.94 ± 0.03
i:DOPC^3	320	13.05 [12.87; 13.23]	59.70 [55.20; 64.81]	3.24 [3.16; 3.35]	11.97 [11.75; 12.20]	21.19 ± 0.04
i:DOPC^h	320	11.86 [11.69; 12.03]	85.13 [71.32; 104.4]	2.97 [2.84; 3.13]	11.02 [10.85; 11.19]	25.01 ± 0.04
i:DOPC^h:AA	298	30.22 [27.00; 34.37]	28.42 [25.32; 32.08]	4.58 [3.95; 5.80]	30.53 [28.16; 33.15]	64.21 ± 0.14
i:DOPC^h:AA	310	28.35 [26.23; 30.92]	28.47 [25.81; 31.50]	4.34 [3.86; 5.14]	25.98 [24.90; 27.11]	57.27 ± 0.08
i:DOPC^h:AA	320	15.00 [14.17; 15.98]	37.96 [32.85; 44.41]	3.40 [3.17; 3.72]	13.04 [12.53; 13.58]	26.65 ± 0.05
i:DPSM^0	320	26.69 [26.46; 26.93]	32.20 [31.58; 32.83]	4.71 [4.54; 4.91]	24.33 [24.14; 24.53]	24.17 ± 0.03
i:DPSM^1	320	25.60 [25.35; 25.86]	34.37 [33.64; 35.11]	4.49 [4.33; 4.66]	23.37 [23.13; 23.61]	31.97 ± 0.06
i:DPSM^2	320	26.51 [26.19; 26.85]	36.66 [35.69; 37.66]	4.31 [4.15; 4.50]	24.35 [24.02; 24.68]	40.80 ± 0.20
i:DPSM^3	320	30.85 [30.42; 31.30]	38.36 [37.40; 39.37]	4.16 [4.02; 4.31]	28.54 [28.07; 29.02]	55.36 ± 0.21
i:DPSM^h	320	36.31 [35.82; 36.81]	41.04 [40.18; 41.92]	3.91 [3.81; 4.01]	34.27 [33.76; 34.78]	72.96 ± 0.16

Table 4: Bending moduli for infinite lipid bilayers calculated using different methods: κ_b^{u}, from undulation spectrum, adjusted to include lipid tilt modulus $\kappa_\theta^{\mathrm{u}}$ and small length scale correction q_c; κ_b^{o}, from orientation spectrum, adjusted to include soft-mode divergence q_c (data not provided); κ_b^{R}, real space fluctuation method. Errors are given as 95% confidence intervals employing parametric bootstrapping ($N = 50,000$ statistically independent samples) assuming Gaussian distributions of the mode-dependent amplitudes[11] (undulation spectrum and orientation spectrum) or standard errors of the mean employing block averaging (at least $N = 8$ independent blocks; real space fluctuations). $*$: Including the divergence term q_c for atomistic DPPC systems does not increase the quality of the fit and is neglected.

System	T in K	R µs^{-1} lipid^{-1}	System	T in K	R µs^{-1} lipid^{-1}
d:DPPC^1	320	2.84 ± 0.09	i:DPPC^1	320	2.50 ± 0.10
d:DPPC^2	320	1.55 ± 0.05	i:DPPC^2	320	1.28 ± 0.04
d:DPPC^3	320	0.79 ± 0.02	i:DPPC^3	320	0.59 ± 0.03
d:DPPC^h	320	0.38 ± 0.02	i:DPPC^h	320	0.27 ± 0.02
			i:DPPC^h:AA	330	< 0.01
d:POPC^1	320	7.51 ± 0.12	i:POPC^1	320	6.85 ± 0.16
d:POPC^2	320	5.71 ± 0.12	i:POPC^2	320	4.94 ± 0.08
d:POPC^2:AA	320	0.49 ± 0.05	i:POPC^3	320	3.89 ± 0.06
d:POPC^3	320	3.55 ± 0.03	i:POPC^4	320	3.48 ± 0.08
d:POPC^h	320	2.84 ± 0.04	i:POPC^h	320	2.42 ± 0.04
d:DOPC^1	320	11.59 ± 0.16	i:DOPC^1	320	10.87 ± 0.16
d:DOPC^2	320	9.17 ± 0.12	i:DOPC^2	320	8.53 ± 0.08
d:DOPC^3	320	6.93 ± 0.08	i:DOPC^3	320	6.12 ± 0.12
d:DOPC^h	320	5.04 ± 0.07	i:DOPC^h	320	4.48 ± 0.06
d:DOPC^h:AA	320	0.41 ± 0.02	i:DOPC^h:AA	298	< 0.01
			i:DOPC^h:AA	310	< 0.01
			i:DOPC^h:AA	320	0.39 ± 0.03
d:DPSM^1	320	3.13 ± 0.10	i:DPSM^1	320	2.54 ± 0.07
d:DPSM^2	320	1.52 ± 0.05	i:DPSM^2	320	1.28 ± 0.04
d:DPSM^3	320	0.72 ± 0.03	i:DPSM^3	320	0.56 ± 0.03
d:DPSM^h	320	0.34 ± 0.01	i:DPSM^h	320	0.24 ± 0.02

Table 5: Cholesterol flipping rates: averaged over all lipids within the bicelle central analysis domain (radius 7 nm) for bicelle systems and averaged over all lipids for infinite lipid bilayer systems. Errors are given as standard errors of the mean employing block averaging (at least $N = 10$ independent blocks). In the original publication of the cholesterol model a value of 4.2 µs^{-1} lipid^{-1} was observed for a 3:1 POPC:CHOL mixture, in excellent agreement with our data[12]. Also other flipping rates for MARTINI membranes fall in the same range: For plasma membrane models rates of 6.53 µs^{-1} lipid^{-1} (310 K)[13] and 5.84 µs^{-1} lipid^{-1} (310 K)[14] were reported. Flipping rates were shown to depend significantly on temperature and lipid saturation (each up to an order of magnitude)[15,16].

**Membrane**	Temperature	Method	κ_b in $k_\mathrm{B}T$	Ref.
**POPC/Chol**		Neutron spin echo (NSE) spectroscopy		
(100 nm LUVs)				
100/0	295		19	(*17*)
90/10	295		20	(*17*)
80/20	295		23	(*17*)
60/40	295		27	(*17*)
50/50	295		37	(*17*)
(200 nm LUVs)				
100/0	298		21	(*18*)
**DOPC/Chol**				
(50 nm LUVs)				
100/0	298		13.01	(*1*)
80/20	298		18.09	(*1*)
70/30	298		23.15	(*1*)
60/40	298		30.31	(*1*)
50/50	298		38.81	(*1*)
(100 nm LUVs)				
100/0	298		19.05	(*1*)
90/10	298		22.46	(*1*)
80/20	298		30.34	(*1*)
100/0	293		20	(*19*)
100/0	293		18-26	(*20*)
**DPPC/Chol**				
(100 nm LUVs)				
100/0	323		46	(*21*)
**POPC/Chol**		X-Ray Scattering		
100/0	303		20.3	(*22*)
100/0	303		19.2,25.7	(*23*)
**DPPC/Chol**				
100/0	323		18.3,27.5	(*23*)
**DOPC/Chol**				
100/0	303		18.2	(*24*)
90/10	303		19.1	(*24*)
80/20	303		17.3	(*24*)
70/30	303		17.7	(*24*)
60/40	303		16.7	(*24*)
90/10	303		16.5	(*25*)
80/20	303		17.2	(*25*)
60/40	303		17.4	(*25*)
100/0	303		16.3,19.4	(*23*)
100/0	303		19.1	(*22*)
100/0	303		19.8	(*26*)
100/0	288		21.4	(*27*)
100/0	303		18.2	(*27*)
100/0	318		16.4	(*27*)

**Membrane**	Temperature	Method	κ_b in $k_\mathrm{B}T$	Ref.
**POPC/Chol**		AFM Indentation		
(40-150$\times 10^3$ nm GUVs)				
100/0	298		38.9	(*28*)
85/15	298		51.0	(*28*)
70/30	298		55.9	(*28*)
55/45	298		63.2	(*28*)
**DOPC/Chol**				
($\approx$150$\times 10^3$ nm GUVs)				
100/0	293		22.2	(*29*)
(Supported Bilayer)				
100/0	293		21.8	(*29*)
**DPPC/Chol**				
($\approx$150$\times 10^3$ nm GUVs)				
100/0	293		383.2	(*29*)
(Supported Bilayer)				
100/0	293		50.2	(*29*)
**POPC/Chol**		Tether Pulling		
100/0	298		16.3	(*30*)
75/25	298		21.2	(*30*)
**DOPC/Chol**				
67/33	295		24.8	(*31*)
**DOPC/Chol**		Micropipette Aspiration		
($\approx$15$\times 10^3$ nm GUVs)				
100/0	294		16	(*32*)
67/33	294		15	(*32*)
(15-30$\times 10^3$ nm GUVs)				
100/0	291		21.2	(*5*)
(20-50$\times 10^3$ nm GUVs)				
67/33	295		21.1	(*31*)
50/50	295		22.3	(*31*)
(10-50$\times 10^3$ nm GUVs)				
100/0	298		11.7,22.8	(*33*)
**POPC/Chol**		Optical Stretching		
($\approx$20$\times 10^3$ nm GUVs)				
100/0	298		8.13	(*34*)
80/20	298		8.5	(*34*)
**DPPC/Chol**				
($\approx$20$\times 10^3$ nm GUVs)				
80/20	298		27.4	(*34*)
**POPC/Chol**		Vesicle Fluctuations		
($\approx$13$\times 10^3$ nm GUV)				
100/0	297		25.6-54.4	(*35*)
($\approx$32$\times 10^3$ nm GUVs)				

**Membrane**	Temperature	Method	κ_b in $k_\mathrm{B}T$	Ref.
100/0	298		35.5	(*36*)
(15-30$\times10^3$ nm GUVs)				
100/0	298		38.5	(*37*)
90/10	298		54.4	(*37*)
80/20	298		70.2	(*37*)
70/30	298		86.8	(*37*)
**DOPC/Chol**				
(13$\times10^3$ nm GUV)				
100/0	297		32.4-43.4	(*35*)
(15-40$\times10^3$ nm GUVs)				
100/0	296		26.4	(*38*)
90/10	296		28.4	(*38*)
80/20	296		27.7	(*38*)
70/30	296		22.5	(*38*)
56/44	296		23.0	(*38*)
50/50	296		26.4	(*38*)
100/0	295		27.3	(*39*)
100/0	295		19,27	(*40*)
(10-50$\times10^3$ nm GUVs)				
100/0	298		≈22	(*41*)
100/0	297		23.2	(*42*)
100/0	295		22.5	(*43*)
100/0	297		21.5	(*44*)
100/0	298		26.8	(*36*)
100/0	298		19.0	(*33*)
**DOPC/Chol**		Electrodeformation		
(15-40$\times10^3$ nm GUVs)				
100/0	296		22.0	(*38*)
90/10	296		20.1	(*38*)
80/20	296		26.2	(*38*)
70/30	296		25.3	(*38*)
**POPC/Chol**		Size Distribution Analysis		
100/0	298		18.5	(*45*)
85/15	298		23.3	(*45*)
71/29	298		28.2	(*45*)
60/40	298		31.1	(*45*)
**DOPC/Chol**				
100/0	298		18.0	(*45*)
85/15	298		23.5	(*45*)
71/29	298		28.1	(*45*)
60/40	298		31.2	(*45*)
**DOPC/Chol**		Time Correlations		
100/0	298		22.1	(*46*)
**DOPC/Chol**		Interferometry		

**Membrane**	Temperature	Method	κ_b in $k_{\mathrm{B}}T$	Ref.
100/0	298		10.5	(*47*)
**POPC/Chol**		Undulations (Martini FF)		
100/0	320		20.9	(*48*)
100/0	300		29.0	(*49*)
**DPPC/Chol**				
100/0	325		15.6	(*50*)
100/0	325		29.9	(*51*)
100/0	325		33.4	(*52*)
100/0	323		35.3	(*53*)
100/0	320		23.0	(*48*)
**DOPC/Chol**				
100/0	300		25.6	(*53*)
80/20	320		21.7	(*53*)
100/0	325		8.55	(*50*)
**POPC/Chol**		Undulations (Coarse-Grained FF)		
100/0	303		13.6	(*54*)
70/30	303		18.2	(*54*)
**DOPC/Chol**				
100/0	303		14.1	(*54*)
70/30	303		11.0	(*54*)
**POPC/Chol**		Undulations (Atomistic FF)		
100/0	300		28.5	(*55*)
70/30	300		74.5	(*55*)
100/0	303		23.3-39.1	(*11*)
**DOPC/Chol**				
100/0	300		30.2	(*55*)
70/30	300		47.8	(*55*)
100/0	298		24.9-42.7	(*11*)
**DPPC/Chol**				
100/0	323		10.2	(*56*)
90/10	323		13.8	(*56*)
85/15	323		15.3	(*56*)
75/25	323		18.8	(*56*)
60/40	323		11.2	(*56*)
100/0	323		27.2-32.6	(*11*)
**POPC/Chol**		Buckling (Martini FF)		
100/0	298		30.3	(*4*)
100/0	323		24.6	(*4*)
**DPPC/Chol**				
100/0	323		29.8	(*4*)
90/10	323		37.2	(*4*)
80/20	323		41.4	(*4*)
70/30	323		56.2	(*4*)
60/40	323		60.7	(*4*)

Membrane	Temperature	Method	κ_b in $k_\mathrm{B}T$	Ref.
100/0	323		32.7	(*57*)
**DOPC/Chol**				
100/0	310		23.4	(*4*)
100/0	323		21.2	(*4*)
80/20	323		26.8	(*4*)
60/40	323		31.6	(*4*)
100/0	323		21.5	(*57*)
100/0	300		37.4	(*57*)
**POPC/Chol**		Density Correlation (Martini FF)		
100/0	320		25.7	(*58*)
**DPPC/Chol**				
100/0	320		29.4	(*58*)
50/50	320		40.8	(*58*)
**DPPC/Chol**		Real Space Fluctuations (RSF) (Martini FF)		
100/0	323		31.9	(*53*)
**DOPC/Chol**				
100/0	300		22.6	(*53*)
80/20	320		21.8	(*53*)
**POPC/Chol**		Real Space Fluctuations (RSF) (CHARMM FF)		
100/0	298		24.3	(*59*)
**DPPC/Chol**				
100/0	323		34.1	(*59*)
80/20	298		130.0	(*59*)
**DOPC/Chol**				
100/0	298		18.3	(*59*)
100/0	298		18.3	(*1*)
90/10	298		22.5	(*1*)
80/20	298		30.5	(*1*)
70/30	298		38.0	(*1*)
60/40	298		52.1	(*1*)
50/50	298		67.7	(*1*)
**POPC/Chol**		Real Space Fluctuations (RSF) (GROMOS FF)		
100/0	298		22	(*60*)
90/10	298		27	(*60*)
70/30	298		50	(*60*)
**DOPC/Chol**		Real Space Fluctuations (RSF) (43A1-S3 FF)		
90/10	303		18	(*61*)
70/30	303		18	(*61*)
**POPC/Chol**		Enhanced Sampling (Multi-Map)		
100/0	300		11.1	(*49*)
70/30	300		21.6	(*49*)
100/0	300		36.2	(*55*)
70/30	300		76.7	(*55*)

**Membrane**	Temperature	Method	κ_b in $k_{\mathrm{B}}T$	Ref.
**DOPC/Chol**				
100/0	300		11.1	(*49*)
70/30	300		15.9	(*49*)
100/0	300		35.1	(*55*)
70/30	300		39.9	(*55*)
**DOPC/Chol**		Enhanced Sampling (Umbrella)		
100/0	310		23.9	(*62*)
**DPPC/Chol**				
100/0	310		29.5	(*62*)
**DPPC/Chol**		Orientation Fluctuations (Martini FF)		
100/0	325		31.2	(*52*)
100/0	325		36.4	(*50*)
100/0	323		34.3	(*53*)
**DOPC/Chol**				
100/0	300		28.9	(*53*)
80/20	320		27.6	(*53*)
100/0	325		26.9	(*50*)
**POPC/Chol**		Orientation Fluctuations (CHARMM FF)		
100/0	303		31.7	(*63*)
100/0	303		23.8,31.8	(*11*)
**DPPC/Chol**				
100/0	323		35.4	(*63*)
100/0	323		35.0	(*64*)
100/0	323		27.5,36.5	(*11*)
**DOPC/Chol**				
100/0	298		28.8	(*63*)
100/0	298		27.7	(*64*)
100/0	298		22.0,29.7	(*11*)

Table 6: Bending modulus κ_b for POPC, DOPC, and DPPC from experiments and simulations in the literature. Values are given in $k_{\mathrm{B}}T$ using the reference temperature provided in the corresponding publication.

Supplementary References

1. S. Chakraborty, M. Doktorova, T. R. Molugu, F. A. Heberle, H. L. Scott, B. Dzikovski, M. Nagao, L. R. Stingaciu, R. F. Standaert, F. N. Barrera, J. Katsaras, G. Khelashvili, M. F. Brown, R. Ashkar, How cholesterol stiffens unsaturated lipid membranes. *Proc. Natl. Acad. Sci. U.S.A.* **117**, 21896–21905 (2020).
2. F. T. Doole, T. Kumarage, R. Ashkar, M. F. Brown, Cholesterol Stiffening of Lipid Membranes. *J. Membr. Biol.* **255**, 385–405 (2022).
3. A. G. Zilman, R. Granek, Undulations and Dynamic Structure Factor of Membranes. *Phys. Rev. Lett.* **77**, 4788–4791 (1996).
4. J. Eid, H. Razmazma, A. Jraij, A. Ebrahimi, L. Monticelli, On Calculating the Bending Modulus of Lipid Bilayer Membranes from Buckling Simulations. *J. Phys. Chem. B* **124**, 6299–6311 (2020).
5. W. Rawicz, K. C. Olbrich, T. McIntosh, D. Needham, E. Evans, Effect of chain length and unsaturation on elasticity of lipid bilayers. *Biophys. J.* **79**, 328–339 (2000).
6. M. Hu, P. Diggins IV, M. Deserno, Determining the bending modulus of a lipid membrane by simulating buckling. *J. Chem. Phys.* **138**, 214110 (2013).
7. G. Khelashvili, B. Kollmitzer, P. Heftberger, G. Pabst, D. Harries, Calculating the bending modulus for multicomponent lipid membranes in different thermodynamic phases. *J. Chem. Theory Comput.* **9**, 3866–3871 (2013).
8. H. Kim, B. Fábián, G. Hummer, Neighbor List Artifacts in Molecular Dynamics Simulations. *ChemRxiv*, 10.26434/chemrxiv-2023–zbj6j (2023).
9. S. Thallmair, M. Javanainen, B. Fábián, H. Martinez-Seara, S. J. Marrink, Nonconverged Constraints Cause Artificial Temperature Gradients in Lipid Bilayer Simulations. *J. Phys. Chem. B* **125**, 9537–9546 (2021).
10. M. Pöhnl, C. Kluge, R. A. Böckmann, Lipid Bicelles in the Study of Biomembrane Characteristics. *J. Chem. Theory Comput.* **19**, 1908–1921 (2023).
11. M. F. Ergüder, M. Deserno, Identifying systematic errors in a power spectral analysis of simulated lipid membranes. *J. Chem. Phys.* **154**, 214103 (2021).
12. M. Melo, H. Ingólfsson, S. Marrink, Parameters for Martini sterols and hopanoids based on a virtual-site description. *J. Chem. Phys.* **143**, 243152 (2015).
13. H. I. Ingólfsson, M. N. Melo, F. J. Van Eerden, C. Arnarez, C. A. Lopez, T. A. Wassenaar, X. Periole, A. H. De Vries, D. P. Tieleman, S. J. Marrink, Lipid organization of the plasma membrane. *J. Am. Chem. Soc.* **136**, 14554–14559 (2014).
14. S. Thallmair, H. I. Ingólfsson, S. J. Marrink, Cholesterol flip-flop impacts domain registration in plasma membrane models. *J. Phys. Chem. Lett.* **9**, 5527–5533 (2018).
15. S. J. Marrink, A. H. De Vries, T. A. Harroun, J. Katsaras, S. R. Wassall, Cholesterol shows preference for the interior of polyunsaturated lipid membranes. *J. Am. Chem. Soc.* **130**, 10–11 (2008).
16. R. X. Gu, S. Baoukina, D. P. Tieleman, Cholesterol Flip-Flop in Heterogeneous Membranes. *J. Chem. Theory Comput.* **15**, 2064–2070 (2019).
17. L. R. Arriaga, I. López-Montero, F. Monroy, G. Orts-Gil, B. Farago, T. Hellweg, Stiffening effect of cholesterol on disordered lipid phases: A combined neutron spin echo + dynamic light scattering analysis of the bending elasticity of large unilamellar vesicles. *Biophys. J.* **96**, 3629–3637 (2009).
18. M. Mell, L. H. Moleiro, Y. Hertle, P. Fouquet, R. Schweins, I. López-Montero, T. Hellweg, F. Monroy, Bending stiffness of biological membranes: What can be measured by neutron spin echo? *Eur. Phys. J. E* **36**, 1–13 (2013).
19. S. Gupta, J. U. De Mel, R. M. Perera, P. Zolnierczuk, M. Bleuel, A. Faraone, G. J. Schneider, Dynamics of Phospholipid Membranes beyond Thermal Undulations. *J. Phys. Chem. Lett.* **9**, 2956–2960 (2018).
20. J. U. De Mel, S. Gupta, R. M. Perera, L. Ngo, P. Zolnierczuk, M. Bleuel, S. V. Pingali, G. J. Schneider, Influence of External NaCl Salt on Membrane Rigidity of Neutral DOPC Vesicles. *Langmuir* **36**, 9356–9367 (2020).
21. M. Nagao, E. G. Kelley, R. Ashkar, R. Bradbury, P. D. Butler, Probing Elastic and Viscous Properties of Phospholipid Bilayers Using Neutron Spin Echo Spectroscopy. *J. Phys. Chem. Lett.* **8**, 4679–4684 (2017).
22. N. Kucerka, S. Tristram-Nagle, J. F. Nagle, Structure of fully hydrated fluid phase lipid bilayers with monounsaturated chains. en, *J. Membr. Biol.* **208**, 193–202 (2005).
23. J. F. Nagle, Experimentally determined tilt and bending moduli of single-component lipid bilayers. *Chem. Phys. Lipids* **205**, 18–24 (2017).
24. J. Pan, T. T. Mills, S. Tristram-Nagle, J. F. Nagle, Cholesterol perturbs lipid bilayers nonuniversally. *Phys. Rev. Lett.* **100**, 198103 (2008).

25. J. C. Mathai, S. Tristram-Nagle, J. F. Nagle, M. L. Zeidel, Structural determinants of water permeability through the lipid membrane. *J. Gen. Physiol.* **131**, 69–76 (2008).
26. M. S. Jablin, K. Akabori, J. F. Nagle, Experimental support for tilt-dependent theory of biomembrane mechanics. *Phys. Rev. Lett.* **113**, 248102 (2014).
27. J. Pan, S. Tristram-Nagle, N. Kucerka, J. F. Nagle, Temperature dependence of structure, bending rigidity, and bilayer interactions of dioleoylphosphatidylcholine bilayers. *Biophys. J.* **94**, 117–124 (2008).
28. D. Drabik, M. Gavutis, R. N. Valiokas, A. R. Ulčinas, Determination of the Mechanical Properties of Model Lipid Bilayers Using Atomic Force Microscopy Indentation. *Langmuir* **36**, 13251–13262 (2020).
29. O. Et-Thakafy, N. Delorme, C. Gaillard, C. Mériadec, F. Artzner, C. Lopez, F. Guyomarc'h, Mechanical Properties of Membranes Composed of Gel-Phase or Fluid-Phase Phospholipids Probed on Liposomes by Atomic Force Spectroscopy. *Langmuir* **33**, 5117–5126 (2017).
30. M. Kocun, A. Janshoff, Pulling tethers from pore-spanning bilayers: towards simultaneous determination of local bending modulus and lateral tension of membranes. *Small* **8**, 847–851 (2012).
31. A. Tian, B. R. Capraro, C. Esposito, T. Baumgart, Bending stiffness depends on curvature of ternary lipid mixture tubular membranes. *Biophys. J.* **97**, 1636–1646 (2009).
32. B. Sorre, A. Callan-Jones, J.-B. Manneville, P. Nassoy, J.-F. Joanny, J. Prost, B. Goud, P. Bassereau, Curvature-driven lipid sorting needs proximity to a demixing point and is aided by proteins. *Proc. Natl. Acad. Sci. U.S.A.* **106**, 5622–5626 (2009).
33. P. Shchelokovskyy, S. Tristram-Nagle, R. Dimova, Effect of the HIV-1 fusion peptide on the mechanical properties and leaflet coupling of lipid bilayers. *New J. Phys.* **13**, 25004 (2011).
34. M. E. Solmaz, S. Sankhagowit, R. Biswas, C. A. Mejia, M. L. Povinelli, N. Malmstadt, Optical stretching as a tool to investigate the mechanical properties of lipid bilayers. *RSC Adv.* **3**, 16632–16638 (2013).
35. D. Drabik, M. Przybyło, G. Chodaczek, A. Iglič, M. Langner, The modified fluorescence based vesicle fluctuation spectroscopy technique for determination of lipid bilayer bending properties. *Biochim. Biophys. Acta* **1858**, 244–252 (2016).
36. Y. Elani, S. Purushothaman, P. J. Booth, J. M. Seddon, N. J. Brooks, R. V. Law, O. Ces, Measurements of the effect of membrane asymmetry on the mechanical properties of lipid bilayers. *Chem. Commun.* **51**, 6976–6979 (2015).
37. J. Henriksen, A. C. Rowat, J. H. Ipsen, Vesicle fluctuation analysis of the effects of sterols on membrane bending rigidity. *Europ. Biophys. J.* **33**, 732–741 (2004).
38. R. S. Gracià, N. Bezlyepkina, R. L. Knorr, R. Lipowsky, R. Dimova, Effect of cholesterol on the rigidity of saturated and unsaturated membranes: Fluctuation and electrodeformation analysis of giant vesicles. *Soft Matter* **6**, 1472–1482 (2010).
39. A. T. Brown, J. Kotar, P. Cicuta, Active rheology of phospholipid vesicles. *Phys. Rev. E* **84**, 021930 (2011).
40. S. A. Rautu, D. Orsi, L. Di Michele, G. Rowlands, P. Cicuta, M. S. Turner, The role of optical projection in the analysis of membrane fluctuations. *Soft Matter* **13**, 3480–3483 (2017).
41. H. A. Faizi, C. J. Reeves, V. N. Georgiev, P. M. Vlahovska, R. Dimova, Fluctuation spectroscopy of giant unilamellar vesicles using confocal and phase contrast microscopy. *Soft Matter* **16**, 8996–9001 (2020).
42. A. I. I. Tyler, J. L. Greenfield, J. M. Seddon, N. J. Brooks, S. Purushothaman, Coupling Phase Behavior of Fatty Acid Containing Membranes to Membrane Bio-Mechanics. *Front. Cell Dev. Biol.* **7**, 187 (2019).
43. D. Kumar, C. M. Richter, C. M. Schroeder, Conformational dynamics and phase behavior of lipid vesicles in a precisely controlled extensional flow. *Soft Matter* **16**, 337–347 (2020).
44. S. Purushothaman, P. Cicuta, O. Ces, N. J. Brooks, Influence of High Pressure on the Bending Rigidity of Model Membranes. *J. Phys. Chem. B* **119**, 9805–9810 (2015).
45. M. A. S. Karal, N. A. Mokta, V. Levadny, M. Belaya, M. Ahmed, M. K. Ahamed, S. Ahammed, Effects of cholesterol on the size distribution and bending modulus of lipid vesicles. *PLoS One* **17**, e0263119 (2022).
46. H. Zhou, B. B. Gabilondo, W. Losert, W. van de Water, Stretching and relaxation of vesicles. *Phys. Rev. E* **83**, 011905 (2011).
47. T. Betz, C. Sykes, Time resolved membrane fluctuation spectroscopy. *Soft Matter* **8**, 5317–5326 (2012).
48. P. Tarazona, E. Chacón, F. Bresme, Thermal fluctuations and bending rigidity of bilayer membranes. *J. Chem. Phys.* **139**, 094902 (2013).
49. G. Fiorin, F. Marinelli, J. D. Faraldo-Gómez, Direct Derivation of Free Energies of Membrane Deformation and Other Solvent Density Variations From Enhanced Sampling Molecular Dynamics. *J. Comput. Chem.* **41**, 449–459 (2020).
50. A. K. Chaurasia, A. M. Rukangu, M. K. Philen, G. D. Seidel, E. C. Freeman, Evaluation of bending modulus of lipid bilayers using undulation and orientation analysis. *Phys. Rev. E* **97**, 1–12 (2018).

51. M. C. Watson, E. S. Penev, P. M. Welch, F. L. Brown, Thermal fluctuations in shape, thickness, and molecular orientation in lipid bilayers. *J. Chem. Phys.* **135** (2011).
52. M. C. Watson, E. G. Brandt, P. M. Welch, F. L. Brown, Determining biomembrane bending rigidities from simulations of modest size. *Phys. Rev. Lett.* **109**, 1–5 (2012).
53. C. Allolio, A. Haluts, D. Harries, A local instantaneous surface method for extracting membrane elastic moduli from simulation: Comparison with other strategies. *Chem. Phys.* **514**, 31–43 (2018).
54. C. M. MacDermaid, H. K. Kashyap, R. H. DeVane, W. Shinoda, J. B. Klauda, M. L. Klein, G. Fiorin, Molecular dynamics simulations of cholesterol-rich membranes using a coarse-grained force field for cyclic alkanes. *J. Chem. Phys.* **143**, 243144 (2015).
55. G. Fiorin, L. R. Forrest, J. D. Faraldo-Gómez, Membrane free-energy landscapes derived from atomistic dynamics explain nonuniversal cholesterol-induced stiffening. *PNAS Nexus* **2**, pgad269 (2023).
56. C. Hofsäß, E. Lindahl, O. Edholm, Molecular dynamics simulations of phospholipid bilayers with cholesterol. *Biophys. J.* **84**, 2192–2206 (2003).
57. D. Bochicchio, L. Monticelli, The membrane bending modulus in experiments and simulations: a puzzling picture. *Adv. Biomembr. Lipid Self-Assem.* **23**, 117–143 (2016).
58. J. Hernández-Muñoz, F. Bresme, P. Tarazona, E. Chacón, Bending Modulus of Lipid Membranes from Density Correlation Functions. *J. Chem. Theory Comput.* **18**, 3151–3163 (2022).
59. M. Doktorova, D. Harries, G. Khelashvili, Determination of bending rigidity and tilt modulus of lipid membranes from real-space fluctuation analysis of molecular dynamics simulations. *Phys. Chem. Chem. Phys.* **19**, 16806–16818 (2017).
60. G. Khelashvili, N. Johner, G. Zhao, D. Harries, H. L. Scott, Molecular origins of bending rigidity in lipids with isolated and conjugated double bonds: The effect of cholesterol. *Chem. Phys. Lipids* **178**, 18–26 (2014).
61. G. Khelashvili, D. Harries, How cholesterol tilt modulates the mechanical properties of saturated and unsaturated lipid membranes. *J. Phys. Chem. B* **117**, 2411–2421 (2013).
62. G. Bubnis, H. J. Risselada, H. Grubmüller, Exploiting Lipid Permutation Symmetry to Compute Membrane Remodeling Free Energies. *Phys. Rev. Lett.* **117**, 188102 (2016).
63. R. M. Venable, F. L. Brown, R. W. Pastor, Mechanical properties of lipid bilayers from molecular dynamics simulation. *Chem. Phys. Lipids* **192**, 60–74 (2015).
64. Z. A. Levine, R. M. Venable, M. C. Watson, M. G. Lerner, J. E. Shea, R. W. Pastor, F. L. Brown, Determination of biomembrane bending moduli in fully atomistic simulations. *J. Am. Chem. Soc.* **136**, 13582–13585 (2014).
