# Supplementary material for: Nonuniversal impact of cholesterol on membranes mobility, curvature sensing and elasticity
Source: Nat Commun. 2023 Dec 11;14:8038. doi: 10.1038/s41467-023-43892-x (PMC10713574; doi:10.1038/s41467-023-43892-x)
Supplement: Supplementary file 3 — Reporting Summary [file 41467_2023_43892_MOESM3_ESM.pdf]

Corresponding author(s): Rainer A. Böckmann

Last updated by author(s): Nov 8, 2023

# Reporting Summary

Nature Portfolio wishes to improve the reproducibility of the work that we publish. This form provides structure for consistency and transparency in reporting. For further information on Nature Portfolio policies, see our Editorial Policies and the Editorial Policy Checklist.

## Statistics

For all statistical analyses, confirm that the following items are present in the figure legend, table legend, main text, or Methods section.

| n/a | Confirmed | |
|---|---|---|
| ☐ | ☒ | The exact sample size (*n*) for each experimental group/condition, given as a discrete number and unit of measurement |
| ☐ | ☒ | A statement on whether measurements were taken from distinct samples or whether the same sample was measured repeatedly |
| ☒ | ☐ | The statistical test(s) used AND whether they are one- or two-sided<br>*Only common tests should be described solely by name; describe more complex techniques in the Methods section.* |
| ☒ | ☐ | A description of all covariates tested |
| ☐ | ☒ | A description of any assumptions or corrections, such as tests of normality and adjustment for multiple comparisons |
| ☐ | ☒ | A full description of the statistical parameters including central tendency (e.g. means) or other basic estimates (e.g. regression coefficient) AND variation (e.g. standard deviation) or associated estimates of uncertainty (e.g. confidence intervals) |
| ☒ | ☐ | For null hypothesis testing, the test statistic (e.g. *F*, *t*, *r*) with confidence intervals, effect sizes, degrees of freedom and *P* value noted<br>*Give P values as exact values whenever suitable.* |
| ☒ | ☐ | For Bayesian analysis, information on the choice of priors and Markov chain Monte Carlo settings |
| ☒ | ☐ | For hierarchical and complex designs, identification of the appropriate level for tests and full reporting of outcomes |
| ☒ | ☐ | Estimates of effect sizes (e.g. Cohen's *d*, Pearson's *r*), indicating how they were calculated |

*Our web collection on statistics for biologists contains articles on many of the points above.*

## Software and code

Policy information about availability of computer code

| | |
|---|---|
| Data collection | Gromacs 2020.1, 2021.4, 2023.1 |
| Data analysis | Python v3.9.5<br>MDAnalysis v1.0.0<br>numpy v1.20.2<br>scipy v1.6.2 |

For manuscripts utilizing custom algorithms or software that are central to the research but not yet described in published literature, software must be made available to editors and reviewers. We strongly encourage code deposition in a community repository (e.g. GitHub). See the Nature Portfolio guidelines for submitting code & software for further information.

## Data

Policy information about availability of data

All manuscripts must include a data availability statement. This statement should provide the following information, where applicable:

- Accession codes, unique identifiers, or web links for publicly available datasets
- A description of any restrictions on data availability
- For clinical datasets or third party data, please ensure that the statement adheres to our policy

All simulation models, input files and structures are available for download from Zenodo [1]. The source data underlying Figures 1b-d, 2a-f, 3a-c, 5 and Supplementary Figures 2, 3a,b, 4a-d, 5a-d, 6a-d, 7a-f, 8a-f, 9-11 are provided as a Source Data file [2].
[1] Pöhnl, M., Trollmann, M. F. & Böckmann, R. A. Non-Universal Impact of Cholesterol on Membranes: Mobility, Curvature Sensing, and Elasticity (Zenodo, 2023). https://doi.org/10.5281/zenodo.10067080.
[2] Pöhnl, M., Trollmann, M. F. & Böckmann, R. A. Source Data for Non-Universal Impact of Cholesterol on Membranes: Mobility, Curvature Sensing, and Elasticity (figshare, 2023). https://doi.org/10.6084/m9. figshare.24518158.

## Research involving human participants, their data, or biological material

Policy information about studies with human participants or human data. See also policy information about sex, gender (identity/presentation), and sexual orientation and race, ethnicity and racism.

| | |
|---|---|
| Reporting on sex and gender | N/A |
| Reporting on race, ethnicity, or other socially relevant groupings | N/A |
| Population characteristics | N/A |
| Recruitment | N/A |
| Ethics oversight | N/A |

Note that full information on the approval of the study protocol must also be provided in the manuscript.

# Field-specific reporting

Please select the one below that is the best fit for your research. If you are not sure, read the appropriate sections before making your selection.

☒ Life sciences ☐ Behavioural & social sciences ☐ Ecological, evolutionary & environmental sciences

For a reference copy of the document with all sections, see nature.com/documents/nr-reporting-summary-flat.pdf

# Life sciences study design

All studies must disclose on these points even when the disclosure is negative.

| | |
|---|---|
| Sample size | For each system we conducted one long simulation. This is sufficient as the study focuses on collective membrane properties (see below), that were determined on a number of independent blocks/windows. Simulation lengths were chosen such as to minimize the statistical errors. |
| Data exclusions | Only equilibrated parts of the trajectories were analyzed. The equilibration time at the start of the simulation was determined by analysis of drift in the respective observable. |
| Replication | Our simulation study focuses on collective membrane properties. Instead of performing several short simulations we instead conducted long simulations (several microseconds) of each system. Reproducibility was ensured through block averaging and parametric bootstrapping for error analysis. Blocks yielded consistent results. |
| Randomization | Randomization is not pertinent in this context, given that the study is based on computational methods only. |
| Blinding | Blinding was not feasible in this case, as the study is entirely computational. |

# Reporting for specific materials, systems and methods

We require information from authors about some types of materials, experimental systems and methods used in many studies. Here, indicate whether each material, system or method listed is relevant to your study. If you are not sure if a list item applies to your research, read the appropriate section before selecting a response.

## Materials & experimental systems

| n/a | Involved in the study |
| --- | --- |
| ☒ | ☐ Antibodies |
| ☒ | ☐ Eukaryotic cell lines |
| ☒ | ☐ Palaeontology and archaeology |
| ☒ | ☐ Animals and other organisms |
| ☒ | ☐ Clinical data |
| ☒ | ☐ Dual use research of concern |
| ☒ | ☐ Plants |

## Methods

| n/a | Involved in the study |
| --- | --- |
| ☒ | ☐ ChIP-seq |
| ☒ | ☐ Flow cytometry |
| ☒ | ☐ MRI-based neuroimaging |

# Plants

Seed stocks

N/A

Novel plant genotypes

N/A

Authentication

N/A
